# The role of social capital in COVID-19 deaths

**DOI:** 10.1186/s12889-021-10475-8

**Published:** 2021-03-03

**Authors:** Janaki Imbulana Arachchi, Shunsuke Managi

**Affiliations:** 1 Department of Civil Engineering, School of Engineering, Kyushu University, 744 Motooka, Nishi-ku, Fukuoka, 819-0395 Japan; 2 Urban Institute, Kyushu University, 744 Motooka, Nishi-ku, Fukuoka, 819-0395 Japan

**Keywords:** Social capital, COVID-19, Deaths, Pandemic

## Abstract

**Background:**

The COVID-19 pandemic has shown a continuously increasing trend with a large variation in the number of COVID-19 deaths across countries. In response, many countries have implemented non pharmaceutical methods of intervention, such as social distancing and lockdowns. This study aims to investigate the relationship of four dimensions of social capital (community attachment, social trust, family bond, and security) and several control variables with COVID-19 deaths.

**Methods:**

We retrieved data from open access databases and a survey. COVID-19 death-related data were collected from the website “Centre for Systems Science and Engineering (CSSE) at Johns Hopkins University”. Social capital-related data were collected from a large-scale survey that included web-based and face-to-face surveys covering 100,956 respondents across all regions/provinces/states of 37 countries in 2017. Data regarding population density, number of hospital beds, and population aged 65 or older were retrieved from the World Development Indicators (WDIs). Data on country lockdowns were obtained from the website “National responses to the 2019-20 coronavirus pandemic”. Linear regressions were applied to identify the relationship between social capital and COVID-19 deaths.

**Results:**

We found that COVID-19 deaths were associated with social capital both positively and negatively. Community attachment and social trust were associated with more COVID-19 deaths, and family bond and security were associated with fewer deaths. COVID-19 deaths were positively associated with population density, ageing population, and interactions between four dimensions of social capital-related factors and the ageing population. Furthermore, the number of hospital beds and early lockdown policy were negatively associated with COVID-19 deaths.

**Conclusions:**

The results indicate that the role of social capital in dynamically evolving threats, such as the current COVID-19 pandemic, is not always negative or positive. Therefore, people’s behaviour should be changed to support countries’ response to the COVID-19 threat.

**Supplementary Information:**

The online version contains supplementary material available at 10.1186/s12889-021-10475-8.

## Background

Since the first report of severe acute respiratory syndrome coronavirus 2 (SARS-CoV-2) in China, coronavirus disease 2019 (COVID-19) [1] has spread as a pandemic affecting the whole world. More than 35 million people were infected, and more than 1 million deaths were reported by October 5, 2020. The COVID-19 pandemic shows a continuously increasing trend and a great variation in infections and deaths across countries. Compared with other countries, most American and European countries have experienced a large number of COVID-19 cases and deaths [2]. In response to the rising numbers of cases and deaths, many countries have implemented non pharmaceutical methods of interventions, such as social distancing, case isolation and quarantine, contact tracing, and lockdowns [3–8]. The methods of controlling the pandemic and the causes behind the differences in cases and deaths among countries have not been identified despite the many related studies. These differences imply that not only clinical characteristics but also social contextual factors, such as social capital, determine COVID-19 deaths.

The concept of social capital was initially defined by sociologists in the 1980s as the aggregated value of connections between individuals and the norms of reciprocity developed from the network [9]. Several definitions of social capital have been advanced, most of which include similar concepts. Social capital is a commonly identified trait of social organization and includes trust between individuals, standards of correspondence and interpersonal connections that could increase the efficiency of society and create platforms that could be beneficial to many parties [10]. We turned to previous studies on social capital and health outcomes to make sense of the emerging relationships between social capital and COVID-19 deaths [11–18]. Although most social capital and health studies have considered the positive side of social capital [11–13, 18], studies related to the negative side of social capital on health have been growing [15–17]. Therefore, the relationship between social capital and health is a double-edged phenomenon [17]. An important problem that needs to be addressed is the connection between social capital and the prevalence of COVID-19-related deaths, based on existing studies on the relationship between social capital and health.

In the pandemic context, clinical studies have shown that COVID-19 mortality can occur due to age, smoking, obesity, lack of immunity, and hospital care, and it is commonly observed among patients with other diseases, such as diabetes and heart diseases [19–22]. However, clinical evidence alone is insufficient to propel the implementation of policies to reduce the number of COVID-19-related deaths, and certain studies related to social capital have attempted to fill the gap in the research on COVID-19. Several studies have concluded that the development and maintenance of different types of social ties influence the response to the COVID-19 pandemic [23]. A recent study analysed the positive association of COVID-19 deaths with social trust [24]. However, previous studies related to COVID-19 have not obtained similar results regarding social capital and COVID-19-related deaths.

This study aims to investigate the relationship of social capital-related factors/variables, including community attachment, social trust, family bond, and security, with several control variables and their influence on COVID-19 deaths, hypothesizing that COVID-19 deaths can be explained more through social capital. Social capital can be measured in different dimensions, and in this study, the four factors of community attachment [25, 26], social trust [25, 27–29], family bond [30, 31], and security [30, 32, 33] were used to measure social capital based on previous studies. Moreover, we assume that relying on both prior pandemic and health studies of the positive relationship between social capital and health [33–35],all four factors may be negatively associated with COVID-19-related deaths. We examined the association of social capital with COVID-19 deaths and additionally considered its association with the aged population and COVID-19 deaths. We further investigated the relationship of COVID-19 deaths with population density, aged population, number of hospital beds, and country lockdown as a proxy for government policy.

## Methods

### Study design and data sources

For this study, we used data from open access databases and a survey. We collected COVID-19-related data from the website “COVID-19 Dashboard by the Centre for Systems Science and Engineering (CSSE) at Johns Hopkins University” [2]. This website has complied data from several important sources, such as the World Health Organization (WHO), European Centre for Disease Prevention and Control (ECDC), and WorldoMeters, which have documented COVID-19 case numbers, death numbers, recovered numbers, active case numbers, testing rate, case-fatality ratio and incidence rate from 188 countries by country and province/state. We identified 35,157,350 COVID-19 cases with 1,037,075 deaths at 11.00 AM on October 05,2020 from the CSSE database.

Social capital data were collected from a large-scale web-based and face-to-face survey of 100,956 respondents across all regions/provinces/states of 37 countries in 2017. The web-based approach was predominantly used in this survey to avoid interviewer bias caused by arbitrary factors. We derived questions in the questionnaire based on the sociology and psychology literature. Additionally, we contacted a survey company in each country to translate the questionnaire into the native language and conduct the survey, and we carefully assessed the surveys to ensure the accuracy of responses through translations and multiple checks by native survey conductors. We used the data of a previous year (2017) instead of during the COVID-19 pandemic to measure social capital in each considered country to gain an understanding of society and people’s behaviour in general conditions. Individuals’ responses change due to unexpected incidents and information. Therefore, we used social capital data collected during stable conditions and checked the influence on unexpected risk events, such as COVID-19 deaths. Data regarding population density, hospital bed numbers, and population aged 65 or older were retrieved from the World Development Indicators (WDIs) [36]. Data on country lockdowns were obtained from the website “National responses to the 2019-20 coronavirus pandemic” [37].

The most recent WDI country data was available for 2018. After merging our survey data with COVID-19 data and WDI country-level data, the study sample consisted of 765,875 deaths in 37 countries. Among these countries, 8 countries, China, India, the USA, Indonesia, Brazil, Russia, Mexico, and Japan, were separated into provinces/states because they had the highest populations in the sample. Therefore, the total number of observations was 294. The sample countries and cumulative COVID-19 deaths are described in Supplementary Table S1.

### Measures

COVID-19 deaths were measured as the number of deaths per square kilometre (km^2^). The deaths per km^2^ were calculated by dividing the number of deaths by the land area km^2^ of 29 countries and the land area km^2^ of the provinces of the 8 most populous countries in the sample.

Social capital was measured by four factors adopted from previous studies, namely, community attachment, social trust, family bond, and security, and were ordinal variables. The four variables were assessed using a single question based on a previous study conducted among the general public [38]. Community attachment is one of the main independent variables in this analysis. The respondents were asked to measure their attachment to the neighbourhood/community in which they currently reside on a 5-point Likert scale, where 1 = not attached at all, 2 = not really attached, 3 = neither attached nor detached, 4 = slightly attached, and 5 = very attached. The average of all items was created, and a higher score indicated that the respondents displayed slight attachment to the community. Social trust is another main variable, and the respondents were asked to measure the importance they attached to “being able to believe people/organizations”. Answers were rated on a 5-point Likert scale, where 1 = not at all important, 2 = not very important, 3 = neither, 4 = somewhat important, and 5 = very important. The average of all items was created, and the high score indicated that to the respondents, social trust was somewhat important. To measure family bond, another main variable, the respondents were asked to measure their feelings about family relationships in the following question: “Do you feel that the relationship with family is important?” A negative answer was rated as 0, and a positive answer was rated as 1. The average of all items was created, and the high score indicated that the respondents considered the family relationship important. For the final main variable, security, the respondents were asked to measure the safety of their neighbourhood according to the question “Do you think the people in your neighbourhood are safe?” Answers were rated on a 5-point Likert scale, where 1 = do not know, 2 = very dangerous, 3 = slightly dangerous, 4 = moderately safe, and 5 = very safe. The average of all items was created, and the high score indicated that respondents considered that the people in their neighbourhood were not safe. For further details, the survey questionnaire is provided in Supplementary Material 2.

Other explanatory variables are population density, population aged 65 or older, the interaction terms between population aged 65 or older and social capital-related factors, number of hospital beds, and country lockdown. Population density was measured by dividing the total population of each country by the land area km^2^ of 29 countries and the land area km^2^ of the provinces of the 8 most populous countries. The population aged 65 or older was measured by dividing the total population of each country by the land area km^2^ of 29 countries and the total population of the provinces of the 8 most populous countries. The number of beds was measured per 1000 people. Country lockdown was a dummy variable measured by the number of days before a shutdown/lockdown decision was made or a stay-at-home order imposed after the first COVID-19 case was reported in China. If a country was locked down or a stay-at-home order was imposed after 50 days, it was called an early lockdown; if it was locked down after 100 days, it was called a late lockdown; and if a country was not locked down, it was referred to as having no lockdown. We used all the variables in log form except the number of beds and country lockdown to make the data conform more closely to the normal distribution and to improve the model fit.

### Statistical analysis

Stata 16 software was used for all analyses. First, we conducted descriptive statistics to describe the centrality of the variables. Second, we conducted Pearson’s correlation analysis to assess the associations between the variables considered in this study. For the correlation analysis, we employed Cohen’s (1992) standard to determine whether the correlation coefficients were substantial, with *r* = .01, .03, and .05 representing small, medium, and large effect sizes, respectively [39]. Finally, we employed multiple linear regression to analyse the influence of various independent variables on COVID-19 deaths, including community attachment, social trust, family bond, and security (to measure social capital), population density, population aged 65 or older, community attachment with population aged 65 or older, social trust with population aged 65 or older, family bond with population aged 65 or older, security with population aged 65 or older, and number of beds and country lockdown. In the multiple regression analyses, COVID-19 deaths per km^2^ were the dependent variable, and the main explanatory variable was social capital. We checked the influence of some independent variables on the dependent variable in this paper. We employed the multiple regression model, an econometric model for testing whether explanatory variables significantly influence the dependent variable, in our analysis. We regressed eight multiple linear regression models with four social capital-related variables, and the other explanatory variables were added one by one. Prior to using multiple regression, simple linear regressions were applied to investigate the correlation between COVID-19 deaths and the four variables used to measure social capital. We divided the sample into two groups: all samples and only the provinces/states of 8 countries. The goal was to examine whether the relationship between COVID-19 deaths and social capital-related variables varied by country. The correlation coefficient and *p*-value of the coefficient for the social capital-related variables were calculated in the analyses of both groups.

## Results

### Descriptive statistics

Table 1 summarizes the means, SDs, medians and ranges of COVID-19 deaths per km^2^ and the regression covariates. For the 294 observations (29 countries and 264 provinces of 8 countries), the mean score for COVID-19 deaths per km^2^ was 0.09 (SD = 0.54), meaning that the score of COVID-19 deaths per km^2^ was relatively low. Regarding the social capital-related factors, the respondents indicated that they were slightly attached to their community (3.71 out of 5), with a median response of 3.64 (IQR 3.38–3.94), and the respondents felt that being able to trust their society was somewhat important (4.53 out of 5), with a median response of 4.57 (IQR 4.40–4.69). Moreover, the respondents reported that they felt that relationships with family were important (0.90 out of 1), with a median response of 0.92 (IQR 0.86–1), and they thought the people in their neighbourhood were slightly dangerous (2.95 out of 5), with a median response of 2.96 (IQR 2.77–3.21). The results for the mean and median of all four factors of social capital-related factors varied little. Furthermore, the mean score of population density was 540.47 (SD = 1793.92); the mean score of the population aged 65 or older per km^2^ was 42.77 (SD = 146.77); and the mean score of the number of beds per 1000 people was 4.28 (SD = 4.21), which was relatively low compared with the mean score ranging from 0.53 to 13.05. However, the country lockdown policy showed that most of the countries decided to shut down/lockdown or impose a stay-at-home order 50 days after the report of the first COVID-19 case in China (2.04 out of 3).

**Table 1:** Descriptive statistics of model variables (*N* = 294)

Variables	Mean	SD	Median	Min	Max
Covid-19 deaths per km^2^	0.09	0.54	0.01	0	7.17
**Social Capital related factors**					
Community attachment (IQR)	3.71	0.53	3.64 (3.38–3.94)	1	5
Social trust (IQR)	4.53	0.30	4.57 (4.40–4.69)	1	5
Family bond (IQR)	0.90	0.21	0.92 (0.86–1)	0	1
Security (IQR)	2.95	0.33	2.96 (2.77–3.21)	1	5
**Other explanatory variables**					
Population density	540.47	1793.9	113	1	19,044
Population aged 65 or older per km^2^	42.77	146.77	6.18	0	1372.60
Community attachment*Population aged 65 or older per km^2^ (Aged65CA)	154.70	557.46	24.45	0	4987.52
Social trust* Population aged 65 or older per km^2^ (Aged65ST)	188.70	656.45	28.58	0	6154.27
Family bond* Population aged 65 or older per km^2^ (Aged65FB)	42.19	158.30	5.47	0	1607.15
Security* Population aged 65 or older per km^2^ (Aged65S)	121.72	421.87	19.16	0	3895.95
Bed number per 1000 people	4.28	4.21	2.22	0.53	13.05
Country lockdown	2.04	0.97	2	1	3

*Note:* Interquartile range in parentheses

### Simple regression analyses: the relationship between COVID-19 deaths per km^2^ and social capital

**Fig. 1:**
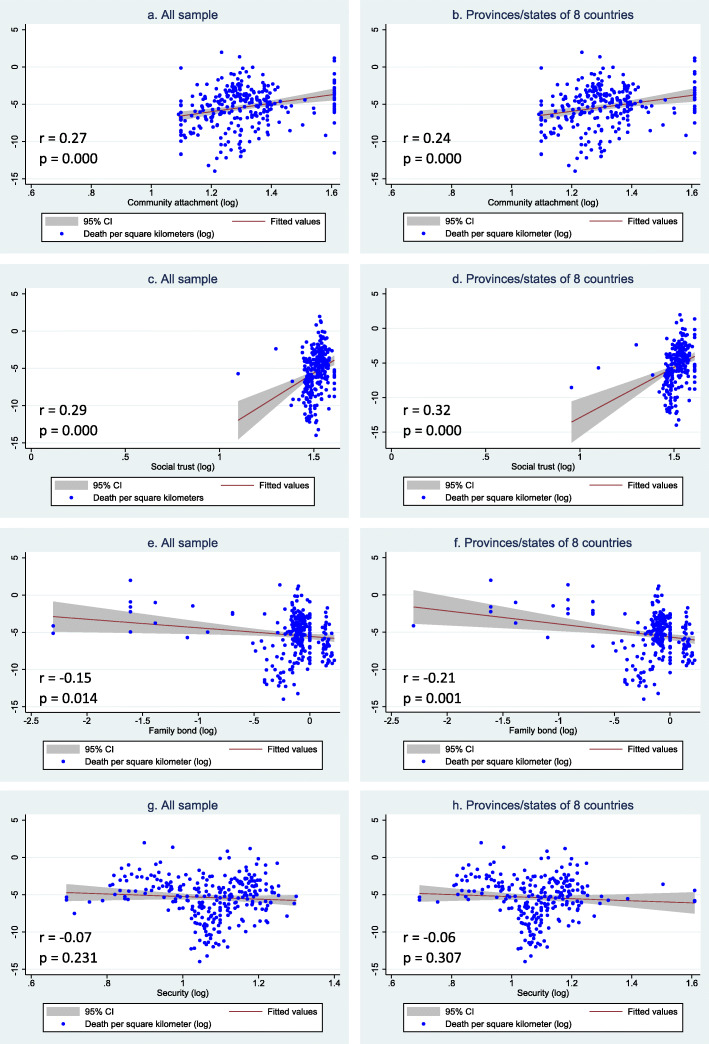
Correlation between COVID-19 deaths per km^2^ and social capital-related factors. The sample was categorized into two groups: **a**, **c**, **e** and **g** refer to 29 countries and the provinces/states of the 8 most populous countries (*N* = 294), and **b**, **d**, **f** and h refer to only the 8 most populous countries by province/state (*N* = 265). Red lines are linear predictions of COVID-19 deaths per km^2^ for each factor of social capital. The 95% confidence intervals of the fitted values are shown in grey (r: correlation coefficient, p: probability value)

The relationship between COVID-19 deaths per km^2^ and social capital-related factors is illustrated in Fig. 1. Figure 1a and b demonstrate that COVID-19 deaths per km^2^ were positively and significantly associated with community attachment for all samples (*r* = 0.27, *p* = 0.000) and for the provinces of 8 countries (*r* = 0.24, *p* = 0.000). Figure 1c and d also displays that the positive correlation between COVID-19 deaths per km^2^ and social trust was significant for all countries (*r* = 0.29, *p* = 0.000) and for the provinces of 8 countries (*r* = 0.32, *p* = 0.000). In contrast, Fig. 1e and f shows a negative and significant correlation between COVID-19 deaths per km^2^ and family bond for all countries (*r* = − 0.15, *p* = 0.014) and for the provinces of 8 countries (*r* = − 0.21, *p* = 0.001). Finally, Fig. 1g and h reveals a negative correlation between COVID-19 deaths per km^2^ and security for all countries (*r* = − 0.07, *p* = 0.231) and for the provinces of 8 countries (*r* = − 0.06, *p* = 0.307).

### Multiple regression analyses

The results of multiple linear regressions for predicting COVID-19 deaths per km^2^ are shown in Table 2. According to our aim, we included social capital-related factors for all specifications (1–8) to check the robustness of the predictions of the social capital-related factors. In columns 1–7, we included the other explanatory variables one-by-one along with the social capital-related factors. In columns 3–6, we included social capital-related factors with the population aged 65 or older as interaction terms. In column 8, we included all explanatory variables simultaneously in the regression model.

**Table 2:** Multiple regression results for COVID-19 deaths

Variables	1	2	3	4	5	6	7	8
Intercept	− 41.974^a^ (6.606)	−33.473^a^ (6.216)	−33.473^a^ (3.552)	− 33.473^a^ (6.216)	−33.473^a^ (6.216)	− 33.473^a^ (6.215)	−19.645^a^ (5.431)	−21.416^a^ (4.623)
Log (Community attachment)	3.767^a^ (1.139)	4.862^a^ (1.019)	4.181^a^ (1.026)	4.862^a^ (1.019)	4.862^a^ (1.019)	4.862^a^ (1.019)	3.581^a^ (1.188)	2.273^b^ (0.997)
Log (Social trust)	21.007^a^ (4.216)	15.859^a^(3.869)	15.859^a^ (3.869)	15.178^a^ (3.855)	15.859^a^(3.869)	15.859^a^ (3.869)	8.285^b^ (3.351)	10.769^a^ (2.831)
Log (Family bond)	−0.858^a^ (0.308)	−1.485^a^ (0.326)	− 1.485^a^ (0.326)	−1.485^a^ (0.326)	−2.166^a^ (0.329)	− 1.485^a^ (0.326)	−0.570^c^ (0.342)	− 0.345^c^ (0.191)
Log (Security)	− 2.983^a^ (0.961)	−3.384^a^ (0.882)	−3.384^a^ (1.035)	− 3.384^a^ (0.882)	−3.384^a^ (0.882)	−4.065^a^ (0.882)	− 3.586^a^ (1.096)	−5.096^a^ (0.849)
Log (population density)	0.623^a^ (0.075)							0.357^a^(0.101)
Log (Aged65 or older)		0.681^a^ (0.049)						0.612^a^(0.087)
Log (Aged65CA)			0.681^a^ (0.049)					
Log (Aged65ST)				0.681^a^ (0.049)				
Log (Aged65FB)					0.681^a^ (0.049)			
Log (Aged65S)						0.681^a^ (0.049)		
Bed number per 1000 people							−0.013 (0.752)	−0.228^a^ (0.036)
Country lockdown								
2 Early lockdowns							−3.843^a^ (0.583)	−1.241 (0.763)
3 Late lockdowns							1.712^a^ (0.331)	0.947^a^ (0.259)
_R_2	0.29	0.52	0.52	0.52	0.52	0.52	0.37	0.67
Adj. R^2^	0.28	0.51	0.51	0.51	0.51	0.51	0.36	0.66
N	278	276	276	276	276	276	278	277

*Note:* The dependent variable was Covid-19 deaths per km^2^ (log). Robust standard error in parentheses. ^a^, ^b^ and ^c^ denote statistical significance at the 99,95 and 90% level, respectively

Among the social capital-related factors, two factors, community attachment and social trust, were associated with more COVID-19 deaths per km^2^. In contrast, two other factors, family bond and security, were associated with fewer COVID-19 deaths per km^2^ in all specifications. Other explanatory variables, population density and population aged 65 or older per km^2^, were associated with more COVID-19 deaths per km^2^; all interaction terms between population aged 65 or older and social capital-related factors were associated with more COVID-19 deaths per km^2^. One additional bed per 1000 people was associated with fewer COVID-19 deaths per km^2^. Early lockdown policies were associated with fewer COVID-19 deaths than late lockdown policy.

### Robustness analyses

As robustness checks, although we included other explanatory variables with social capital-related factors in several multiple regressions, the sign of the influence of social capital-related factors on COVID-19 deaths was not changed. Furthermore, the robust standard error implies that there was no heteroscedasticity, and diagnostic tests confirmed normality and no multicollinearity in the regressions. In addition, we tested the correlation between COVID-19 deaths per km^2^ and social capital-related factors by the 8 most populous countries separately. Most of the results were similar to those in Fig. 1. The results are presented in Supplementary Fig. S1.

## Discussion

To the best of our knowledge, this is the first systematic study to examine the impact of social capital on COVID-19 deaths. Multiple regression analysis revealed that, as we hypothesized, COVID-19 deaths are associated with social capital-related factors in two dimensions. Community attachment and social trust were associated with increased COVID-19 deaths, while family bond and security, were associated with reduced COVID-19 deaths in this study. The key findings of the study are discussed below.

In this study, we found that a one percentage point increase in average community attachment and social trust are associated with a 4% and a 14% (on average) increase in COVID-19 deaths, respectively. In contrast, a one percentage point increase in average family bond and security are associated with a 1% and a 4% (on average) reduction in COVID-19 deaths, respectively. Therefore, the positive impact of social capital-related factors was larger than the negative impact on COVID-19 deaths in all specifications. In terms of correlation coefficients, similar conclusions were observed at the country level as well as at the province level for 8 countries (Fig. 1). Although we expected to find negative associations, given the prior evidence of a positive link between social capital and health [e.g., [12–14, 18]], community attachment and social trust were positively associated with COVID-19 deaths. These findings are consistent with the fact that a low level of institutional trust interferes with endeavours to contain transmission through physical distancing [40], and societies with high social trust might be more vulnerable to deception about the severity of COVID-19, counterfeit treatments, and contemptuous perspectives on physical distancing [41]. In addition, family bond and security were negatively associated with COVID-19 deaths. These findings align with previous findings that family social capital has several dimensions, with its components showing a clear relationship with health [31], and that neighbourhood social capital influences health communication [32, 33, 42]. These results suggest that social capital is a double-edged phenomenon [17] and does not always positively or negatively affect health [24].

Population density was found in this study to be associated with more COVID-19 deaths per km^2^, which supports previous study findings that the population density is associated with the COVID-19 outbreak and related deaths [43–45]. Recent clinical studies have observed that older people have a high COVID-19 mortality risk [19, 21, 22]. Our study also confirmed that a high population aged 65 or older was significantly associated with high COVID-19 deaths. In addition, the interaction terms of social capital-related factors with the population aged 65 or older appeared to be associated with more deaths from COVID-19. The number of hospital beds was negatively and significantly associated with COVID-19 deaths. This finding implies that the hospital bed is a critical input in treating COVID-19-infected patients [46]. In addition, the early lockdown policy was a more effective response reducing COVID-19 deaths than the no lockdown and late lockdown policies [7, 47].

## Strengths and limitations

There are some limitations in this study. First, this study included only 37 countries based on our survey data. However, our sample included the countries reporting the highest COVID-19 infections and deaths, including the USA, India, Brazil, Russia, and Spain. Second, we selected only a limited number of factors that potentially determine COVID-19 deaths in a country. To improve the prediction accuracy, other factors may also be included in future studies. Finally, although COVID-19 data were available at the country level as well as at the province level, there was a lack of public data for other demographic variables in certain countries. Additional weaknesses in this study include the limited sample size. It is important to cover many countries when conducting a global analysis, even though we covered 37 countries with different cultures and demographic characteristics. Furthermore, social capital-related factors can change over time, and the use of more years of data can help identify those changes, while our study addresses only the current state of social capital in the considered countries. Even though this study has come limitations and weaknesses, it covers 37 countries with a significant number of observations and different demographic characteristics. Furthermore, while many previous studies have discussed social capital in different dimensions, social capital has not been investigated with regard to COVID-19. Therefore, the results of this study can contribute to future pandemic-related policymaking at the country level.

## Conclusion

In response to the rising numbers of COVID-19 cases and deaths, most countries have implemented interventions to control the pandemic until a vaccine is developed. It is important to identify social contextual factors associated with the health impacts of COVID-19 to support public health policy.

In summary, our analysis found that social capital-related factors were associated with COVID-19 deaths in two dimensions. Community attachment and social trust were associated with more COVID-19-related deaths, while family bond and security were associated with fewer COVID-19-related deaths. In addition, higher COVID-19 deaths were associated with higher population density, an ageing population, fewer hospital beds, and lower government effectiveness. Social capital-related factors show both positive and negative effects on COVID-19 deaths, demonstrating a dynamic role of social capital and indicating that social capital does not always affect health positively or negatively. From this analysis, we conclude that social determinants of health are significantly affected by dynamically evolving threats, such as the current COVID-19 pandemic. People’s behaviours should be changed to support countries’ response to the COVID-19 threat.

## Supplementary Information

**Additional file 1.**

**Additional file 2.**

## Data Availability

This study is used both open access data and survey data. For Covid-19 deaths related data, we used the dataset maintained by the Centre for Systems Science and Engineering (CSSE) at Johns Hopkins University: https://coronavirus.jhu.edu/map.html. Data regarding population density, hospital beds numbers, and population age 65 or older, was retrieved from the World Development Indicators: https://datatopics.worldbank.org/world-development-indicators/. Data on country lockdown was obtained from the website “National responses to the 2019-20 coronavirus pandemic”: https://en.wikipedia.org/wiki/National_responses_to_the_COVID-19_pandemic. Social capital data was collected from a large-scale survey of 100,956 respondents across 37 countries that including web-based and face-to-face surveys covering all regions/provinces/states of 37 countries in 2017. Survey data is available from the corresponding author on reasonable request.

## Abbreviations

COVID-19: Coronavirus disease 2019
SARS-CoV-2: Severe acute respiratory syndrome coronavirus 2
CSSE: Centre for systems science and engineering
WHO: World health Organization
ECDC: European centre for disease prevention and control
WDIs: World development indicators
SD: Standard deviation
IQR: Interquartile range
