# Supplementary material for: The role of social capital in COVID-19 deaths
Source: BMC Public Health. 2021 Mar 3;21:434. doi: 10.1186/s12889-021-10475-8 (PMC7928173; doi:10.1186/s12889-021-10475-8)
Supplement: Supplementary file 1 — Additional file 1. [file 12889_2021_10475_MOESM1_ESM.docx]

**The role of social capital on Covid-19 deaths**

Janaki Imbulana Arachchi^1^, Shunsuke Managi^1,2,*^

Department of Civil Engineering, School of Engineering, Kyushu University, 744 Motooka, Nishi-ku, Fukuoka 819-0395, Japan

2 Urban Institute, Kyushu University, 744 Motooka, Nishi-ku, Fukuoka 819-0395, Japan

* Corresponding author. Department of Civil Engineering, School of Engineering, Kyushu University, 744 Motooka, Nishi-ku, Fukuoka 819-0395, Japan, [managi@doc.kyushu-u.ac.jp](mailto:managi@doc.kyushu-u.ac.jp)

**Supplementary Material 1**

**Table S1.** Cumulative Covid-19 deaths of 37 countries (as of October 5, 2020)

| Country | Covid-19 deaths | Country | Covid-19 deaths | Country | Covid-19 deaths |
| --- | --- | --- | --- | --- | --- |
| Thailand | 58 | Hungary | 642 | Maine | 138 |
| Malaysia | 128 | Poland | 2203 | Maryland | 3861 |
| Singapore | 27 | Czech | 465 | Massachusetts | 9260 |
| Vietnam | 35 | Romania | 4185 | Michigan | 6955 |
| Philippines | 4630 | Sri Lanka | 13 | Minnesota | 1994 |
| Venezuela | 494 | **USA** | **197001** | Mississippi | 2780 |
| Chile | 12013 | Alabama | 2401 | Missouri | 1782 |
| Colombia | 22924 | Alaska | 44 | Montana | 143 |
| South Africa | 15499 | Arizona | 5409 | Nebraska | 442 |
| Myanmar | 32 | Arkansas | 1166 | Nevada | 1506 |
| Kazakhstan | 1634 | California | 14812 | New Hampshire | 438 |
| Mongolia | 0 | Colorado | 2006 | New Jersey | 16057 |
| Egypt | 5661 | Connecticut | 4488 | New Mexico | 836 |
| Australia | 816 | Delaware | 619 | New York | 33070 |
| Germany | 9356 | Florida | 13086 | North Carolina | 3180 |
| UK | 41726 | Georgia | 6474 | North Dakota | 184 |
| France | 30958 | Hawaii | 107 | Ohio | 4580 |
| Spain | 29848 | Iowa | 1250 | Oklahoma | 930 |
| Italy | 35624 | Idaho | 434 | Oregon | 521 |
| Sweden | 5846 | Illinois | 8624 | Pennsylvania | 7893 |
| Netherland | 6296 | Indiana | 3478 | Rhode Island | 1085 |
| Greece | 310 | Kansas | 582 | South Carolina | 3158 |
| Canada | 9217 | Kentucky | 1093 | South Dakota | 193 |
| Turkey | 7119 | Louisiana | 5313 | Tennessee | 2164 |

| Texas | 14826 | Gansu | 2 | Goa | 327 |
| --- | --- | --- | --- | --- | --- |
| Utah | 437 | Tibet | 0 | Gujarat | 3270 |
| Vermont | 58 | Macao | 0 | Haryana | 1069 |
| Virginia | 2918 | Jiangxi | 1 | Himachal Pradesh | 98 |
| Washington | 2031 | Jiangsu | 0 | Jammu & Kashmir | 951 |
| West Virginia | 297 | Guangxi Zhuang | 2 | Jharkhand | 590 |
| Wisconsin | 1230 | Shanghai | 7 | Karnataka | 7629 |
| Wyoming | 49 | Liaoning | 2 | Kerala | 489 |
| Washington, D.C. | 619 | Hebei | 6 | Madhya Pradesh | 1877 |
| **China** | **4737** | Shanxi | 0 | Maharashtra | 31351 |
| Beijing | 9 | Tianjin | 3 | Manipur | 51 |
| Jilin | 2 | Xinjiang Uygur | 3 | Meghalaya | 31 |
| Hunan | 4 | Hubei | 4512 | Mizoram | 0 |
| Sichuan | 3 | Shaanxi | 3 | Nagaland | 15 |
| Chongqing | 6 | Qinghai | 0 | Odisha | 669 |
| Fujian | 1 | Ningxia Hui | 0 | Puducherry | 431 |
| Guangdong | 8 | Henan | 22 | Punjab | 2646 |
| Guizhou | 2 | **India** | **83236** | Rajasthan | 1293 |
| Hainan | 6 | Andhra Pradesh | 5177 | Tamil Nadu | 8618 |
| Zhejiang | 1 | Arunachal Pradesh | 13 | Tripura | 228 |
| Heilongjiang | 13 | Assam | 528 | Uttar Pradesh | 4771 |
| Anhui | 6 | Bihar | 855 | Uttarak hand | 460 |
| Inner Mongolia | 1 | Chandigarh | 109 | West Bengal | 4183 |
| Hong Kong | 103 | Chhattisgarh | 628 | **Indonesia** | **9460** |
| Shandong | 7 | Dadra & Nagar Havel | 2 | Aceh | 130 |
| Yunnan | 2 | Delhi | 4877 | Bali | 206 |

| Bangka-Belitung | 4 | South Sumatera | 322 | Santa Catarina | 2609 |
| --- | --- | --- | --- | --- | --- |
| Banten | 124 | North Sumatera | 394 | Sergipe | 1968 |
| Bengkulu | 31 | Yogyakarta | 54 | São Paulo | 33472 |
| Gorontalo | 69 | **Brazil** | **134935** | Tocantins | 840 |
| Jakarta | 1527 | Acre | 646 | **Russia** | **19489** |
| Jambi | 8 | Alagoas | 2002 | Volga | 872 |
| West Java | 311 | Amazonas | 3931 | Central | 7669 |
| Central Java | 1243 | Amapa | 688 | Ural | 1146 |
| East Java | 2942 | Bahia | 6132 | North Caucasus | 2062 |
| West Kalimantan | 7 | Ceara | 8774 | East Siberian | 1057 |
| South Kalimantan | 396 | Distrito Federal | 3022 | West Siberian | 1332 |
| Central Kalimantan | 127 | Espírito Santo | 3399 | Volga-Vyatka | 840 |
| East Kalimantan | 265 | Goias | 3995 | Northwestern | 3019 |
| South Sulawesi | 389 | Maranhao | 3622 | Central Black Earth | 350 |
| Riau Island | 96 | Minas Gerais | 6500 | Far Eastern | 462 |
| Lampung | 28 | Mato Grosso do Sul | 1133 | Northern | 612 |
| Maluku | 38 | Mato Grosso | 3178 | Northern | 68 |
| North Maluku | 72 | Para | 6421 | Kaliningrad | 872 |
| West Nusa Tenggara | 182 | Paraiba | 2670 | **Mexico** | **66329** |
| East Nusa Tenggara | 5 | Pernambuco | 7954 | Aguascalientes | 432 |
| Papua | 72 | Piaui | 2007 | Baja California | 3196 |
| Riau | 96 | Paraná | 4018 | Baja California Sur | 368 |
| West Sulawesi | 7 | Rio de Janeiro | 17453 | Campeche | 775 |
| Central Sulawesi | 12 | Rio Grande do Norte | 2333 | Coahuila de Zaragoza | 1452 |
| South east Sulawesi | 46 | Rondo Nia | 1289 | Colima | 447 |
| North Sulawesi | 167 | Roraima | 611 | Chiapas | 1005 |
| West Sumatera | 90 | Rio Grande do Sul | 4268 | Chihuahua | 1169 |

| Distrito Federal | 10725 | Hokkaido | 106 | Shiga | 7 |
| --- | --- | --- | --- | --- | --- |
| Durango | 462 | Aomori | 1 | Kyoto | 25 |
| Guanajuato | 2231 | Iwate | 0 | Osaka | 187 |
| Guerrero | 1681 | Miyagi | 2 | Hyogo | 55 |
| Hidalgo | 1658 | Akita | 0 | Nara | 9 |
| México | 8170 | Yamagata | 1 | Wakayama | 4 |
| Michoacán de Acampo | 1243 | Fukushima | 0 | Tottori | 0 |
| Morelos | 987 | Ibaraki | 16 | Shimane | 0 |
| Nayarit | 610 | Tochigi | 1 | Okayama | 1 |
| Nuevo León | 2362 | Gunma | 19 | Hiroshima | 3 |
| Oaxaca | 1260 | Saitama | 97 | Yamaguchi | 1 |
| Puebla | 3620 | Chiba | 67 | Tokushima | 6 |
| Querétaro Arteaga | 765 | Tokyo | 389 | Kagawa | 2 |
| Quintana Roo | 1416 | Kanagawa | 131 | Ehime | 6 |
| San Luis Potosí | 1227 | Niigata | 0 | Kochi | 3 |
| Sinaloa | 2840 | Toyama | 25 | Fukuoka | 88 |
| Sonora | 2678 | Ishikawa | 44 | Saga | 0 |
| Tabasco | 2620 | Fukui | 8 | Nagasaki | 3 |
| Tamaulipas | 1850 | Yamanashi | 5 | Kumamoto | 8 |
| Tlaxcala | 928 | Nagano | 1 | Oita | 2 |
| Veracruz Llave | 3739 | Gifu | 10 | Miyazaki | 1 |
| Yucatán | 1350 | Shizuoka | 2 | Kagoshima | 12 |
| Zacatecas | 521 | Aichi | 77 | Okinawa | 45 |
| **Japan** | **1474** | Mie | 4 |  |  |

*Note:* Total number of countries are 37 and China, India, USA, Indonesia, Brazil, Russia, Mexico, and Japan were separated by provinces. Data for Covid-19 death collected from the Coronavirus COVID-19 global cases by the Johns Hopkins university website.

**Table S 2.** Questions and answers to measures social capital related factors

| Variables | Questions | Answers |
| --- | --- | --- |
| Community attachment | How attached are you to your local community? | 5.Completely attached  4.Slightly attached  3.Neither  2.Slightly detached  1.Completely detached |
| Social trust | To be believed by people/organizations is | 5.Very important  4.Somewhat important  3.Neither  2.Not very important  1.Not at all important |
| Family bond | Relationship with family is important or not in your life? | 1.Important  0.Not important |
| Security | Please tell us about safety of your neighborhood. | 5.Very safe  4.Moderately safe  3.Slightly dangerous  2.Very dangerous  1.Do not know |

*Note:* Part of survey questions in 2017

**Figure S1.** Correlations between social capital related factors and Covid-19 deaths per km^2^ of 8 counties by provinces and rest of other 29 countries

Supplementary figure S1 illustrated that further correlations between social capital related factors and Covid-19 deaths per km^2^ each country by provinces. The most of results were shown similar trend with both regressions and figure 1 results.


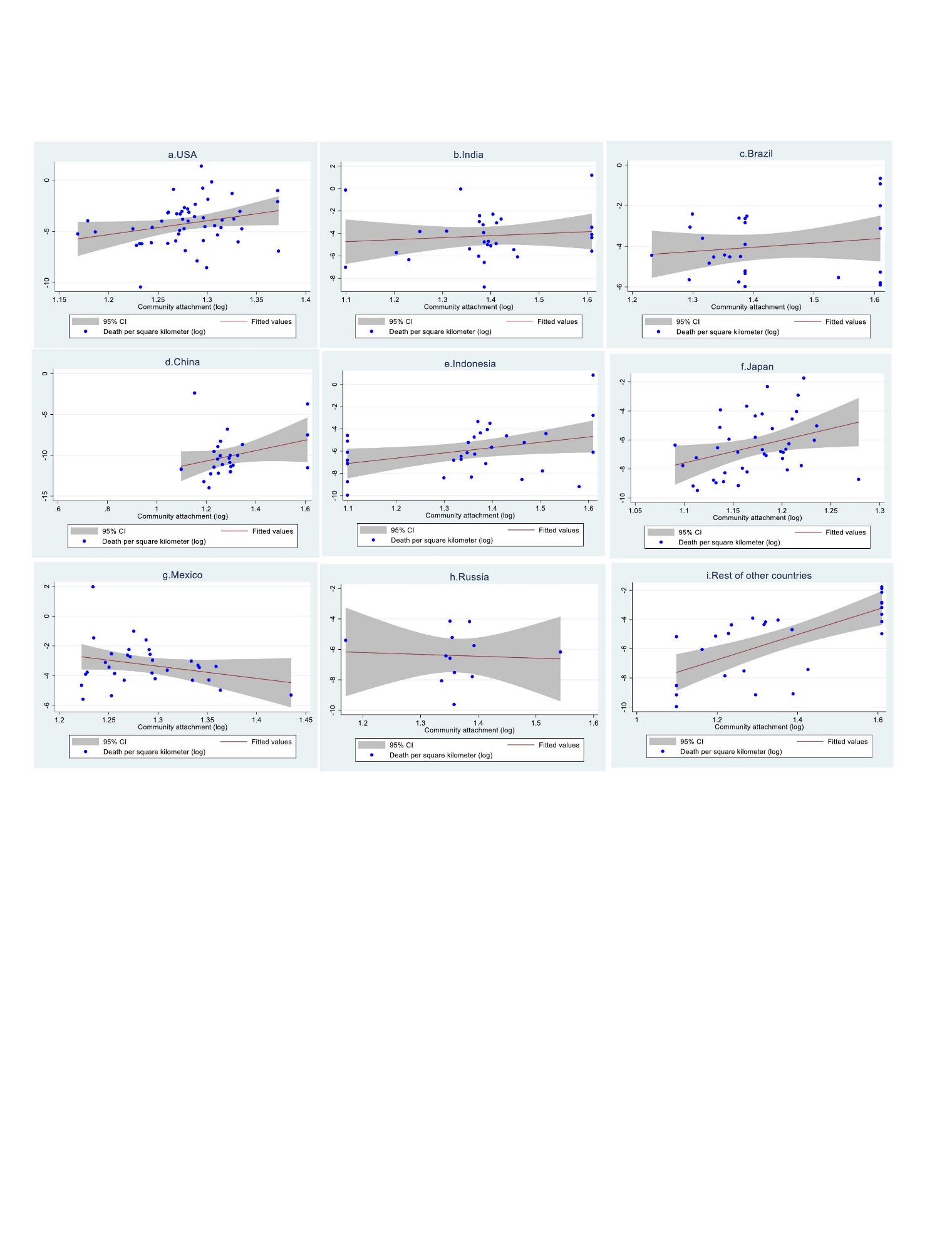


**Figure S1.1.** Correlations between community attachment and Covid-19 deaths per km^2^ of 8 counties by provinces and rest of other 29 countries. Red lines are linear predictions of Covid-19 deaths per km^2^ on average community attachment. The 95% confidence intervals of the fitted values are shown by grey areas


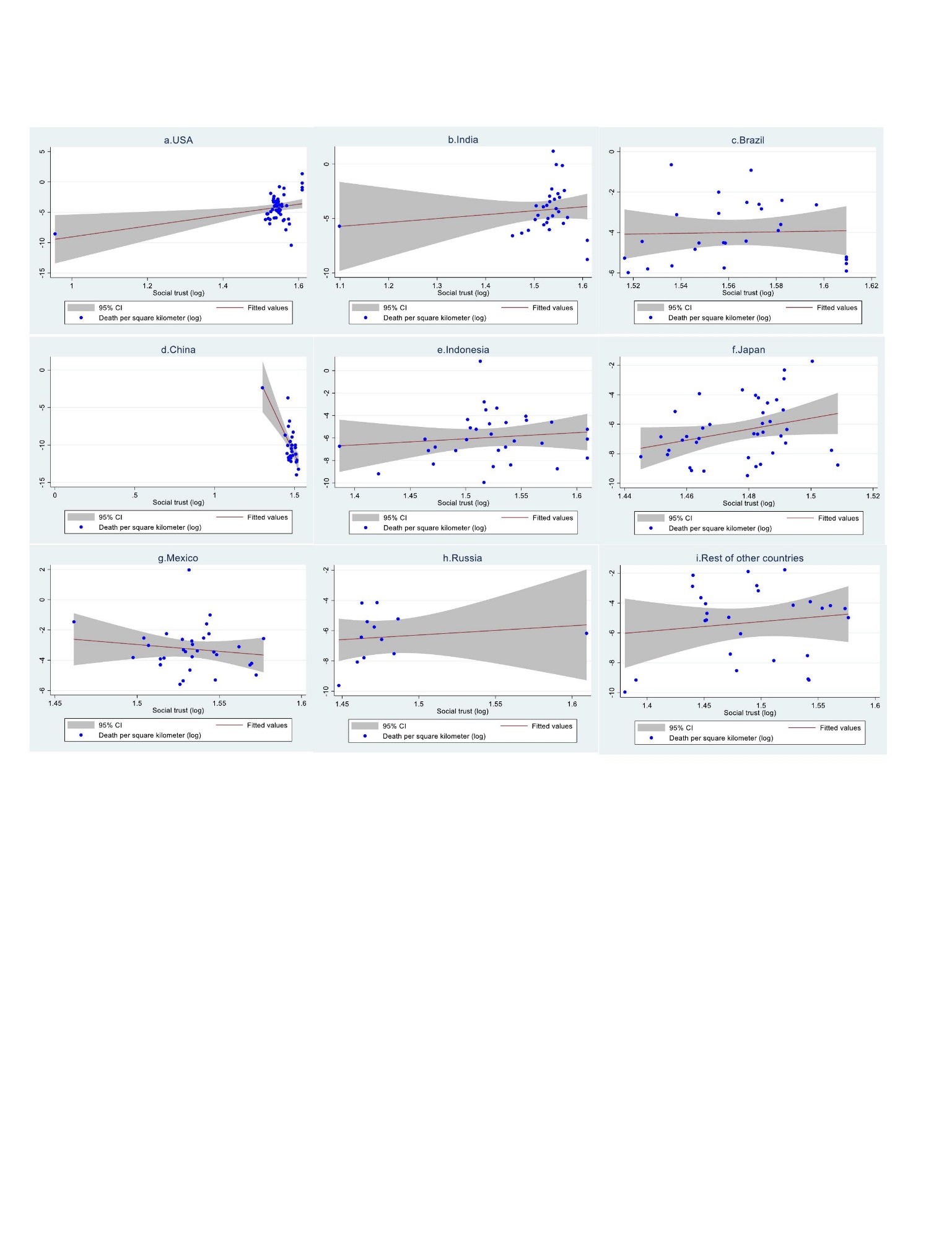


**Figure S1.2.** Correlations between social trust and Covid-19 deaths per km^2^ of 8 counties by provinces and rest of other 29 countries. Red lines are linear predictions of Covid-19 deaths per km^2^ on average social trust. The 95% confidence intervals of the fitted values are shown by grey areas


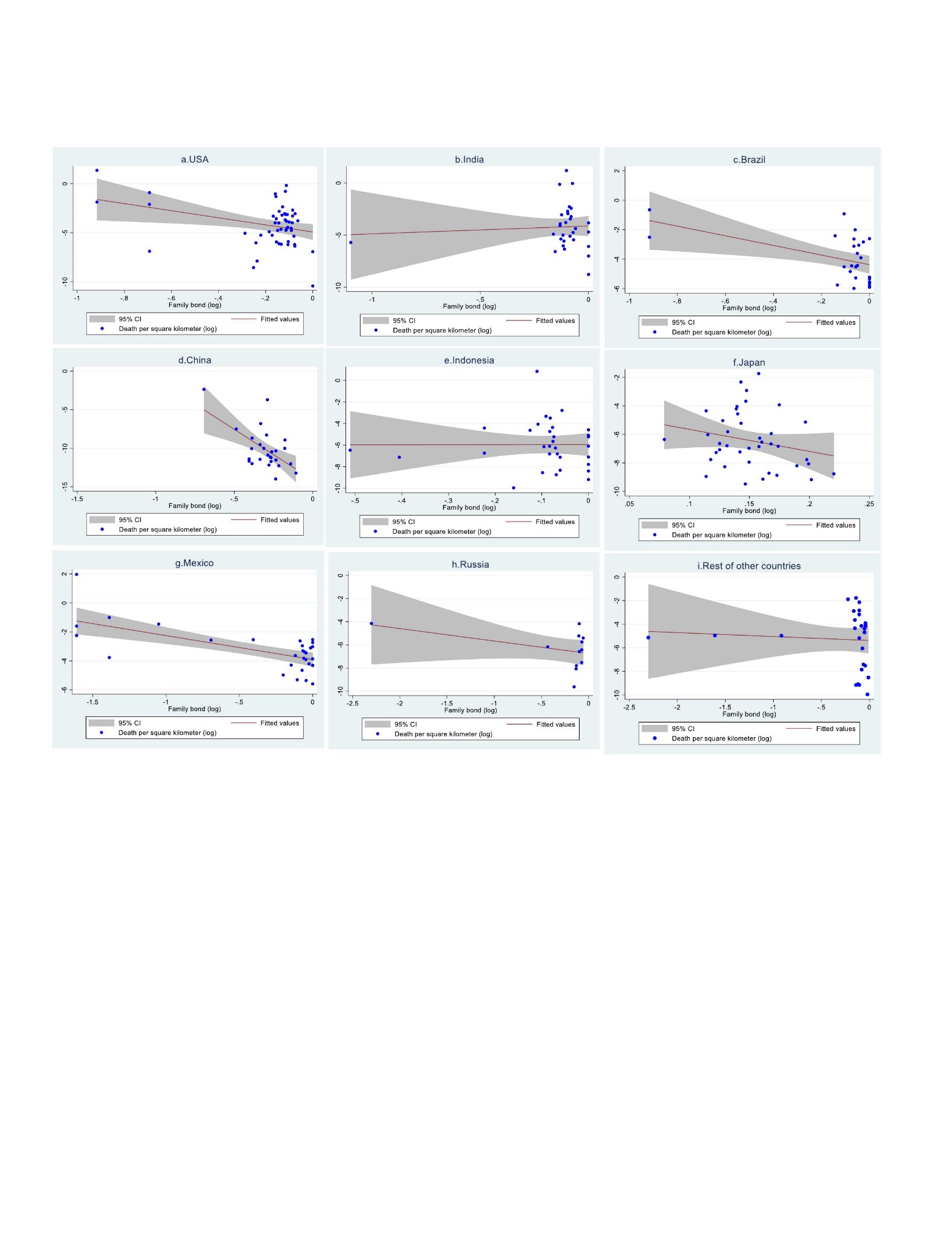


**Figure S1.3.** Correlations between Family bond and Covid-19 deaths per km^2^ of 8 counties by provinces and rest of other 29 countries. Red lines are linear predictions of Covid-19 deaths per km^2^ on average family bond. The 95% confidence intervals of the fitted values are shown by grey areas


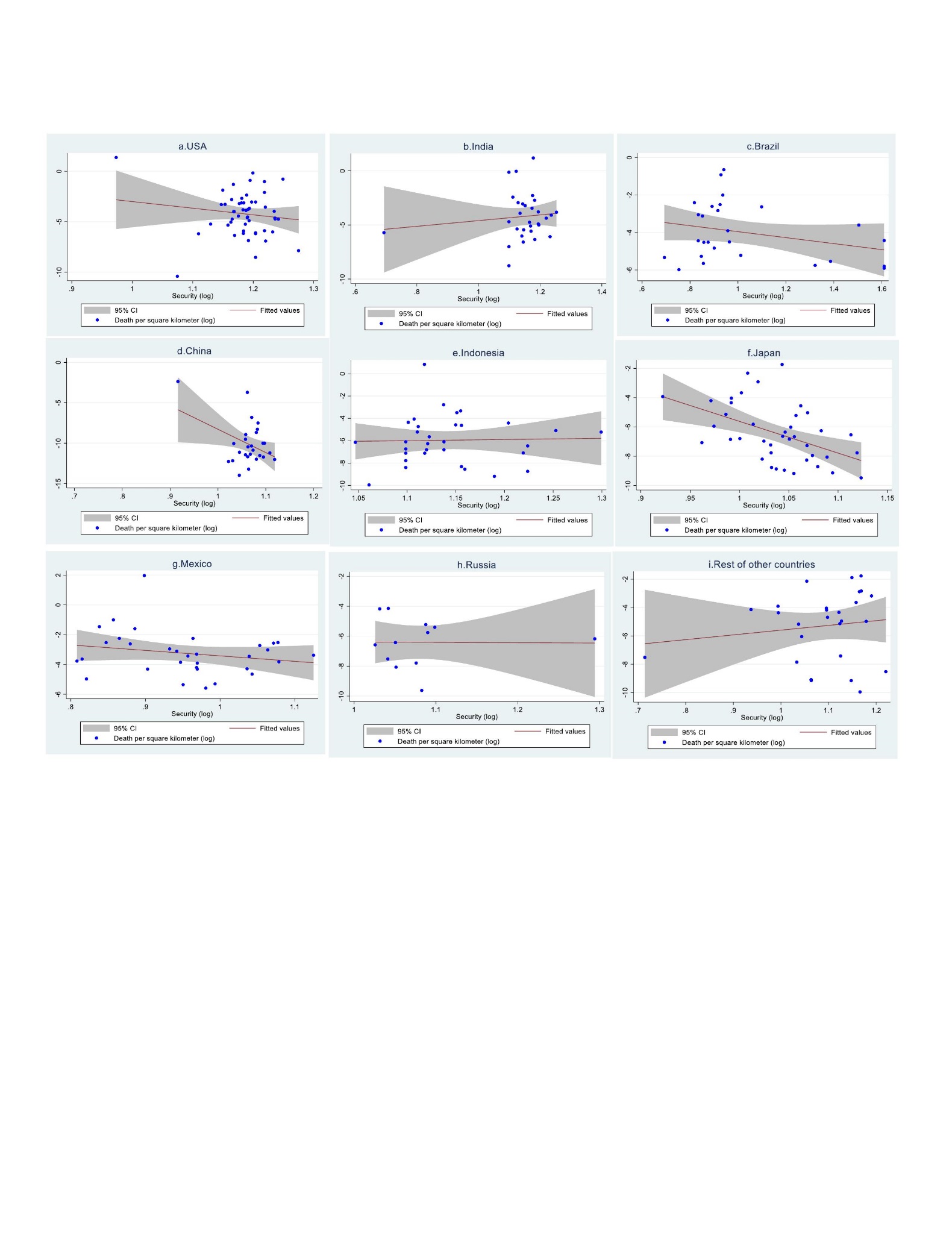


**Figure S1.4.**Correlations between Security and Covid-19 deaths per km^2^ of 8 counties by provinces and rest of other 29 countries. Red lines are linear predictions of Covid-19 deaths per km^2^ on average security. The 95% confidence intervals of the fitted values are shown by grey areas
